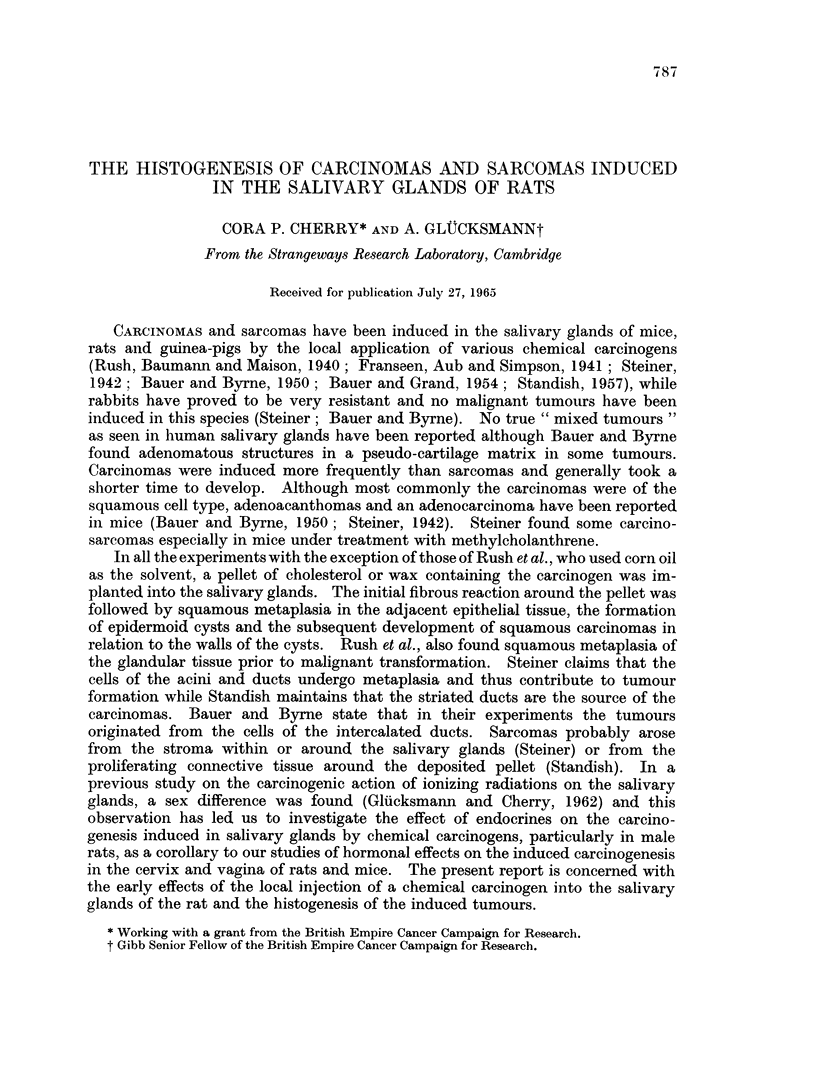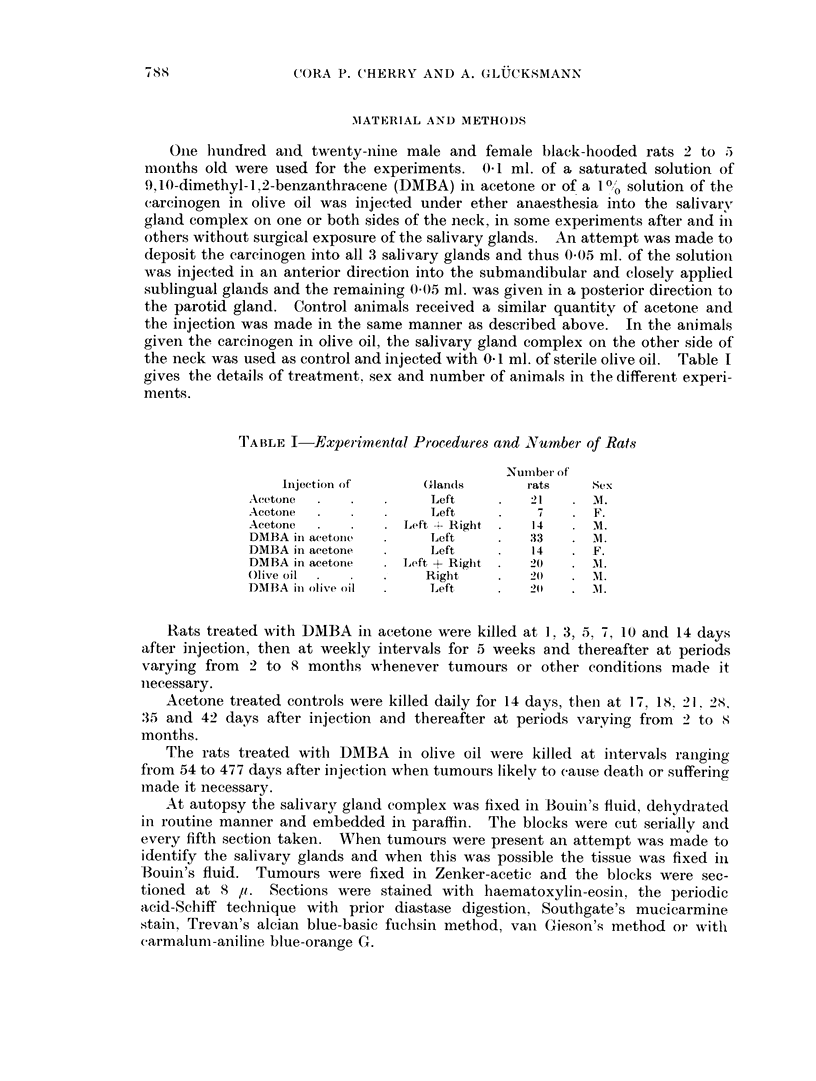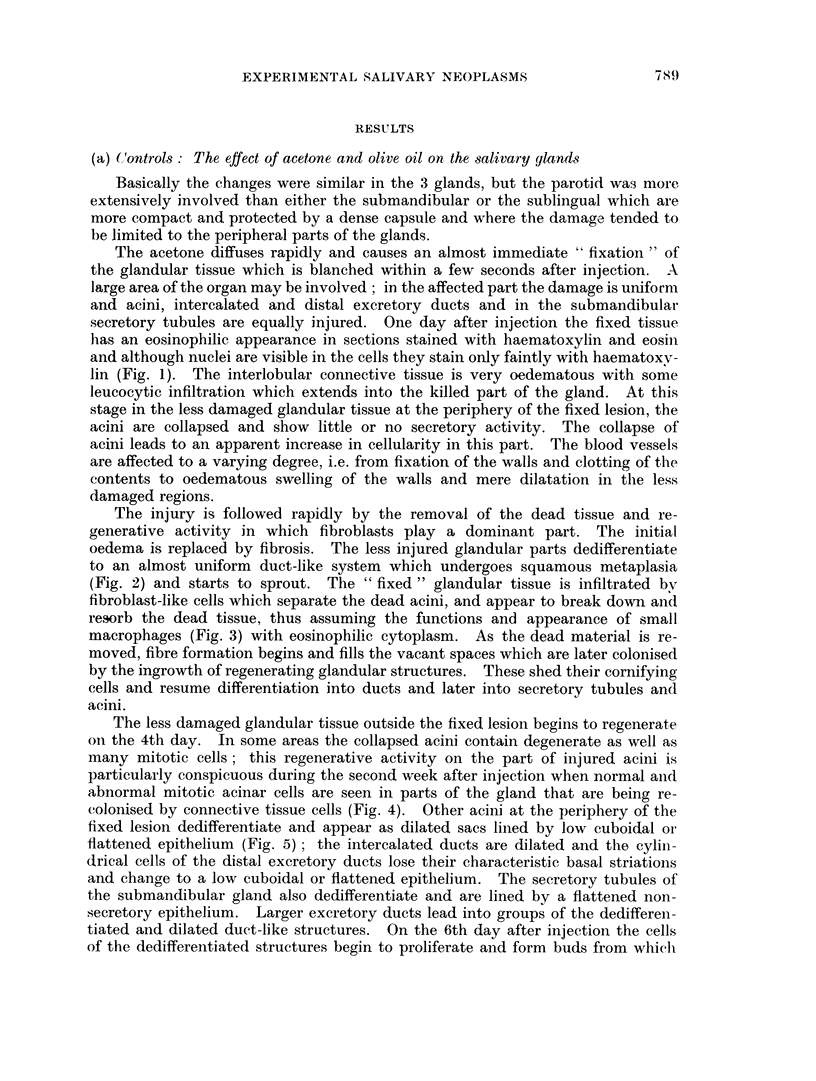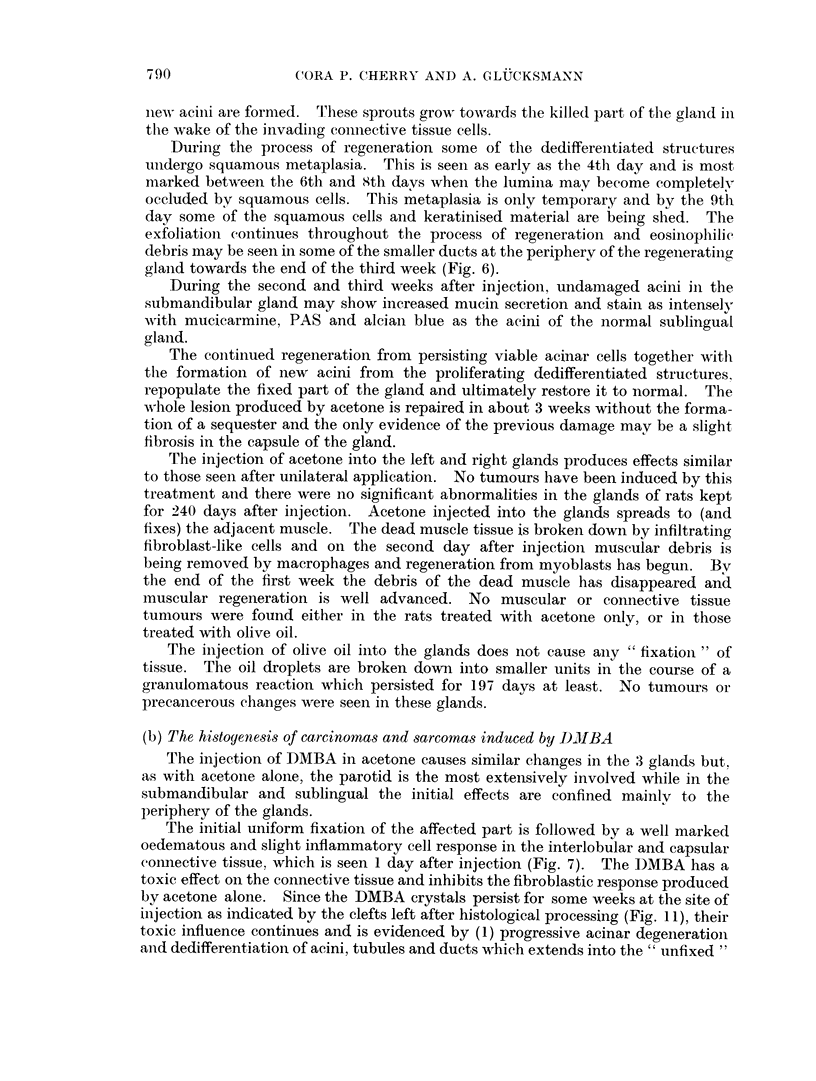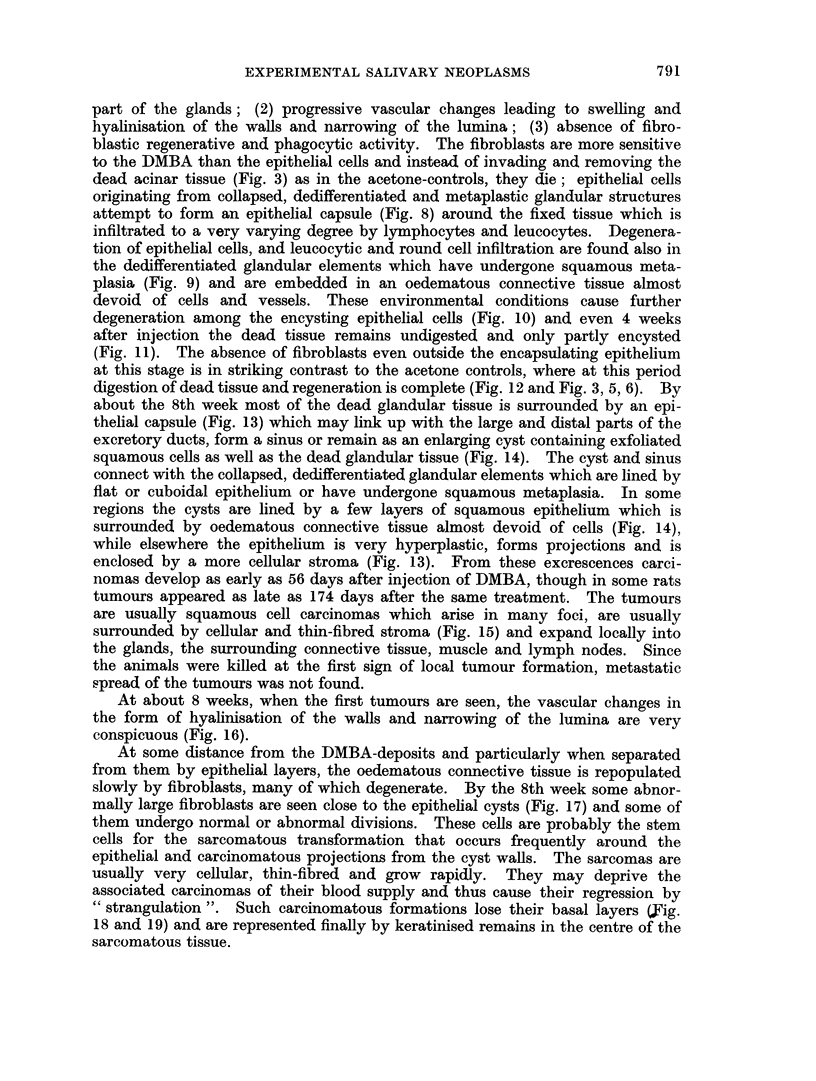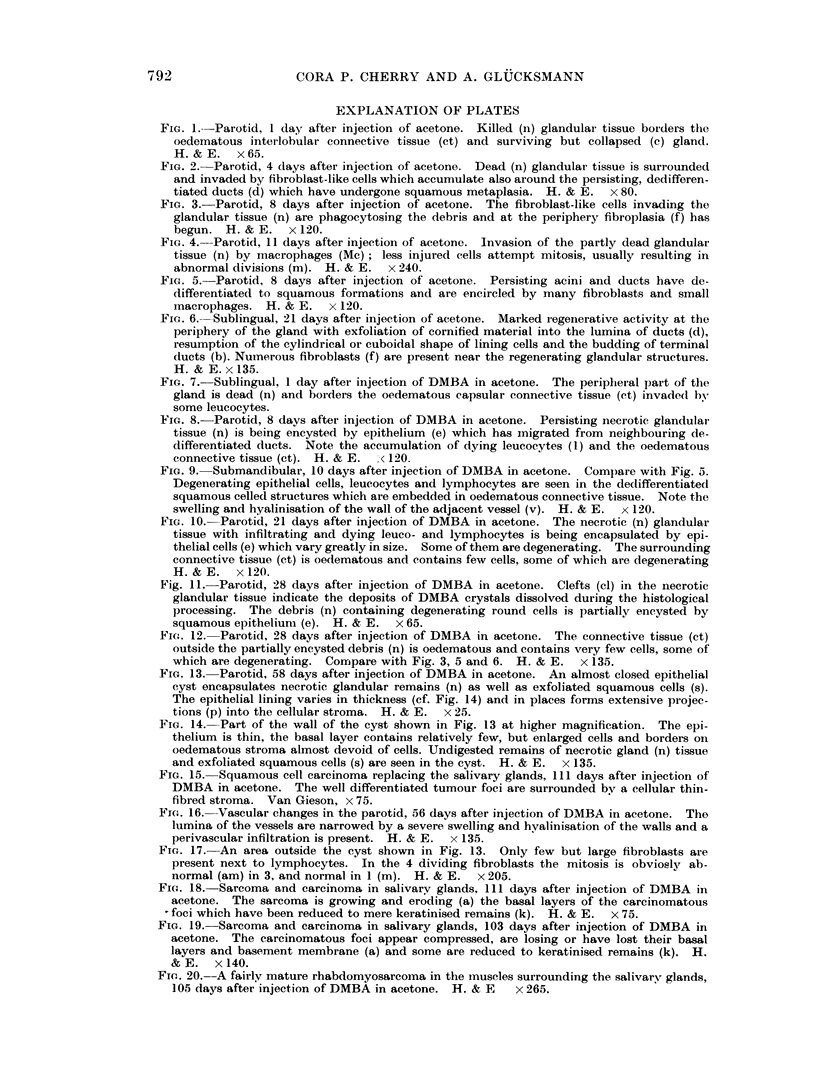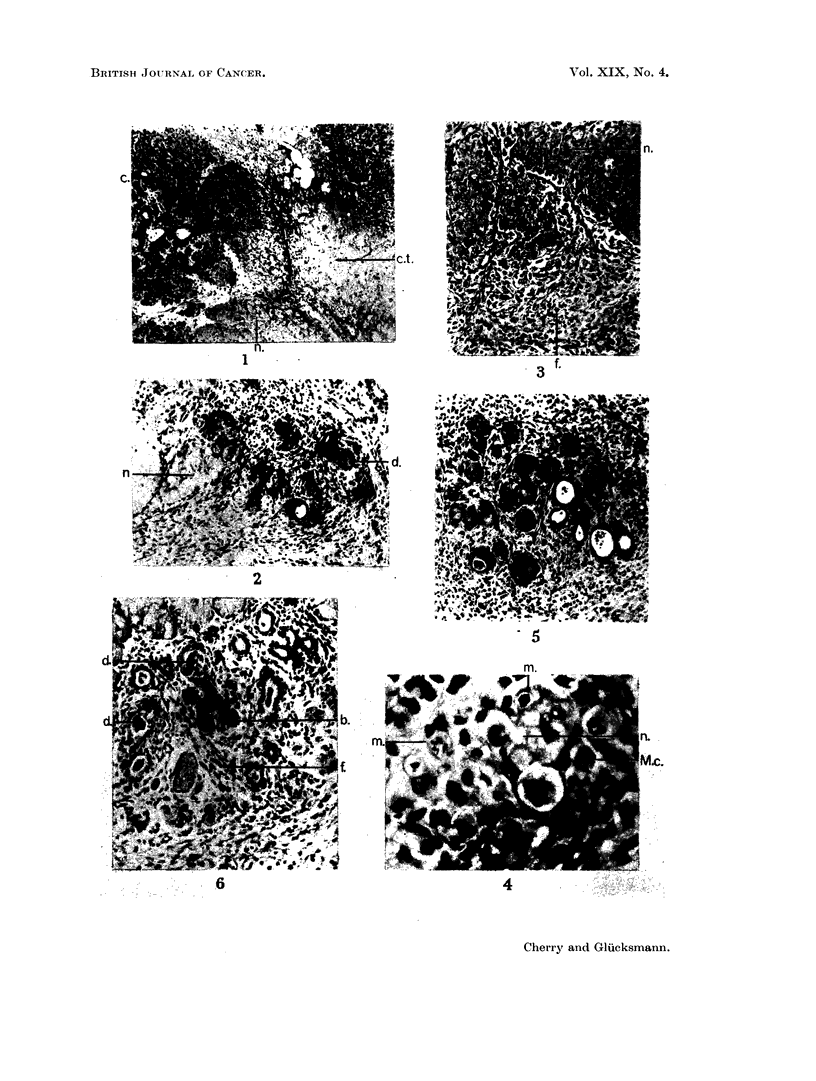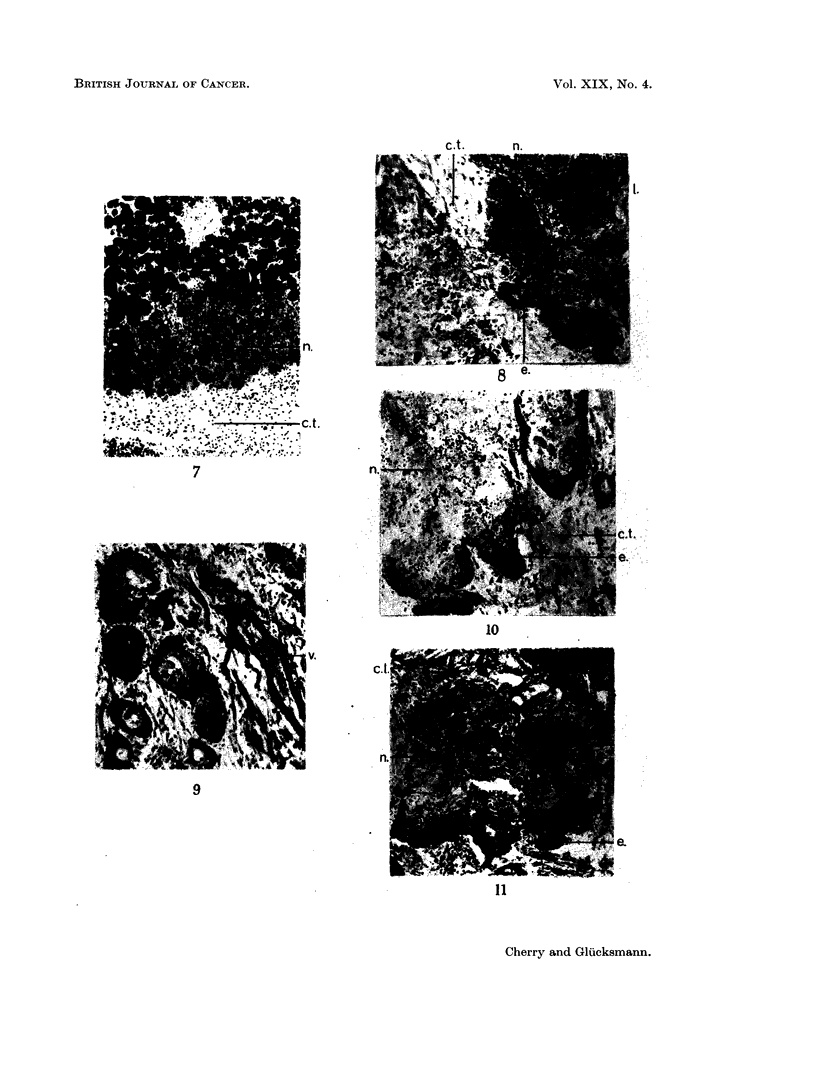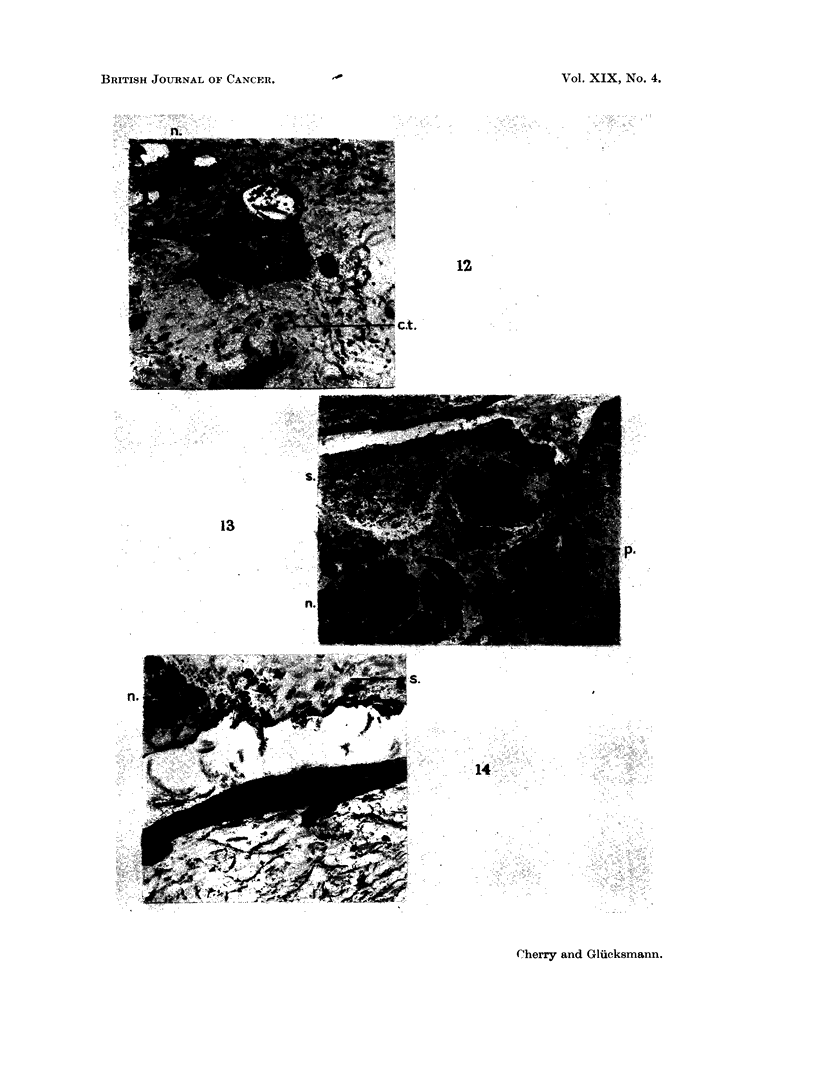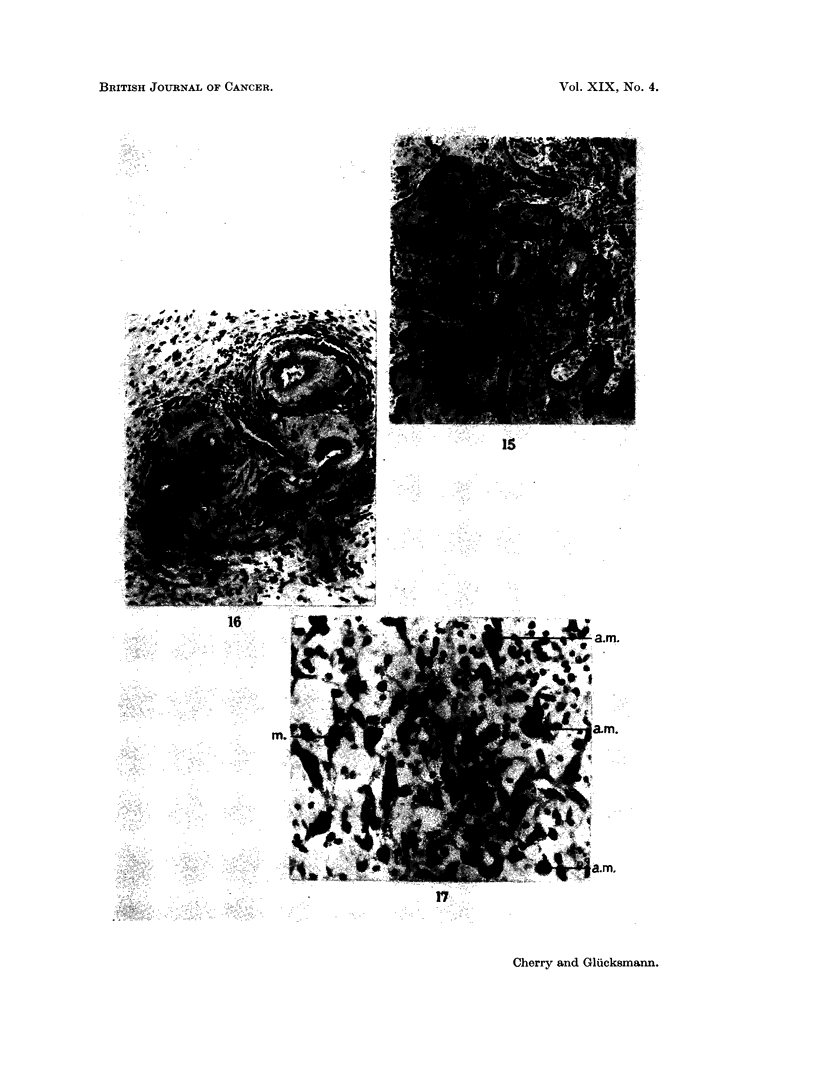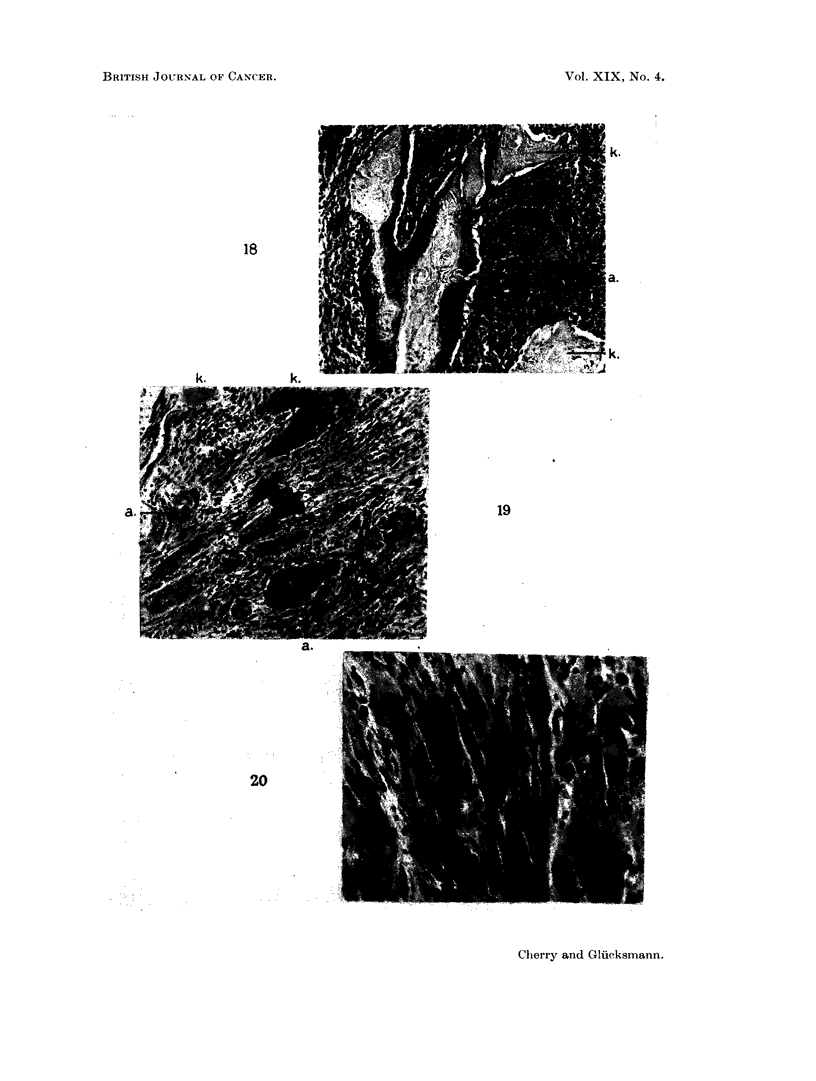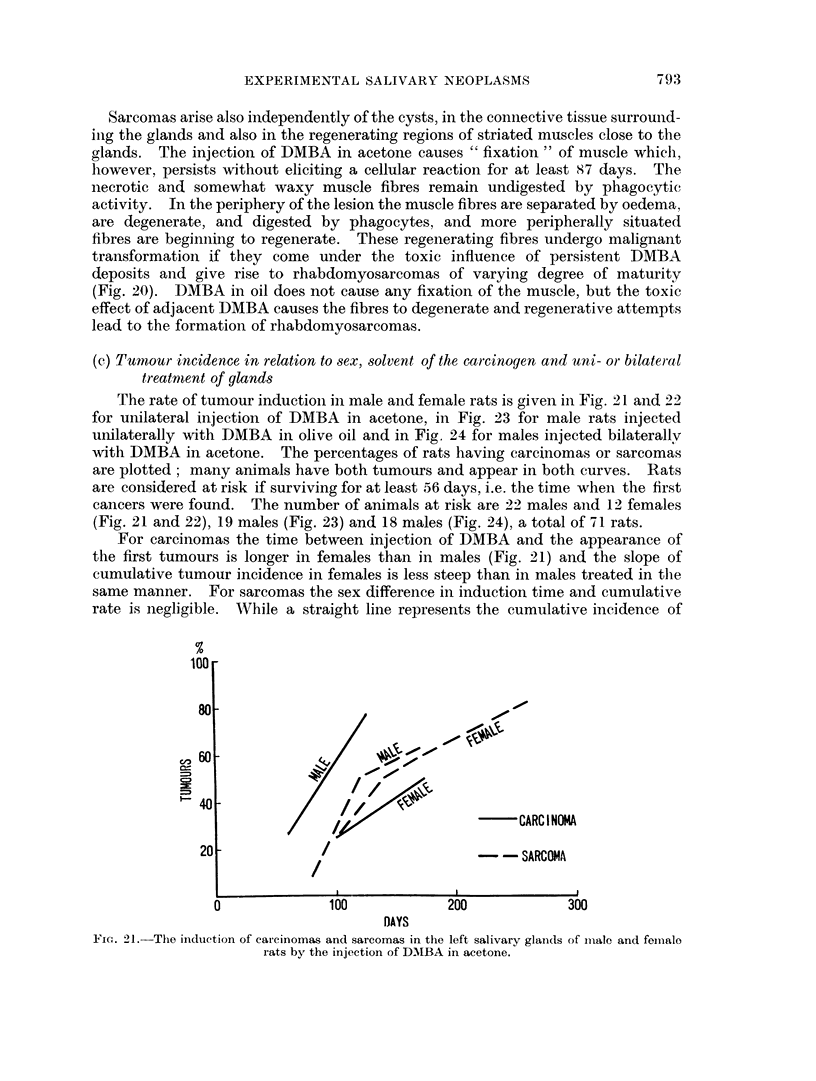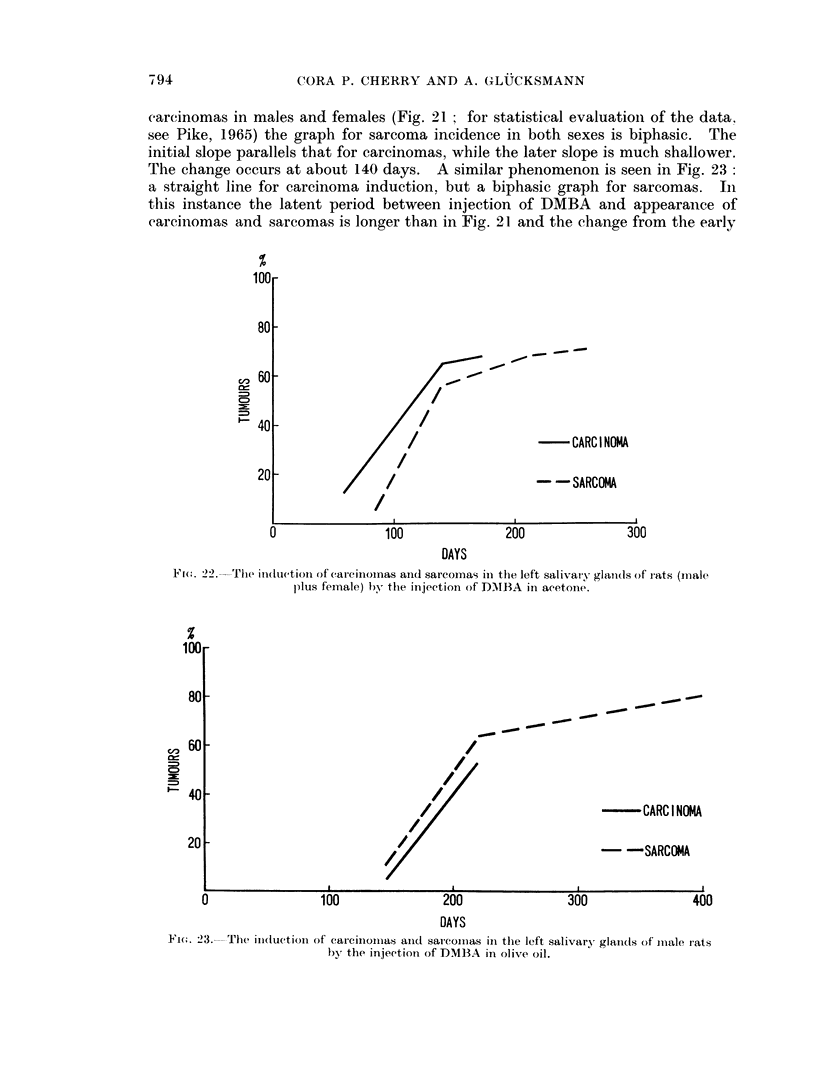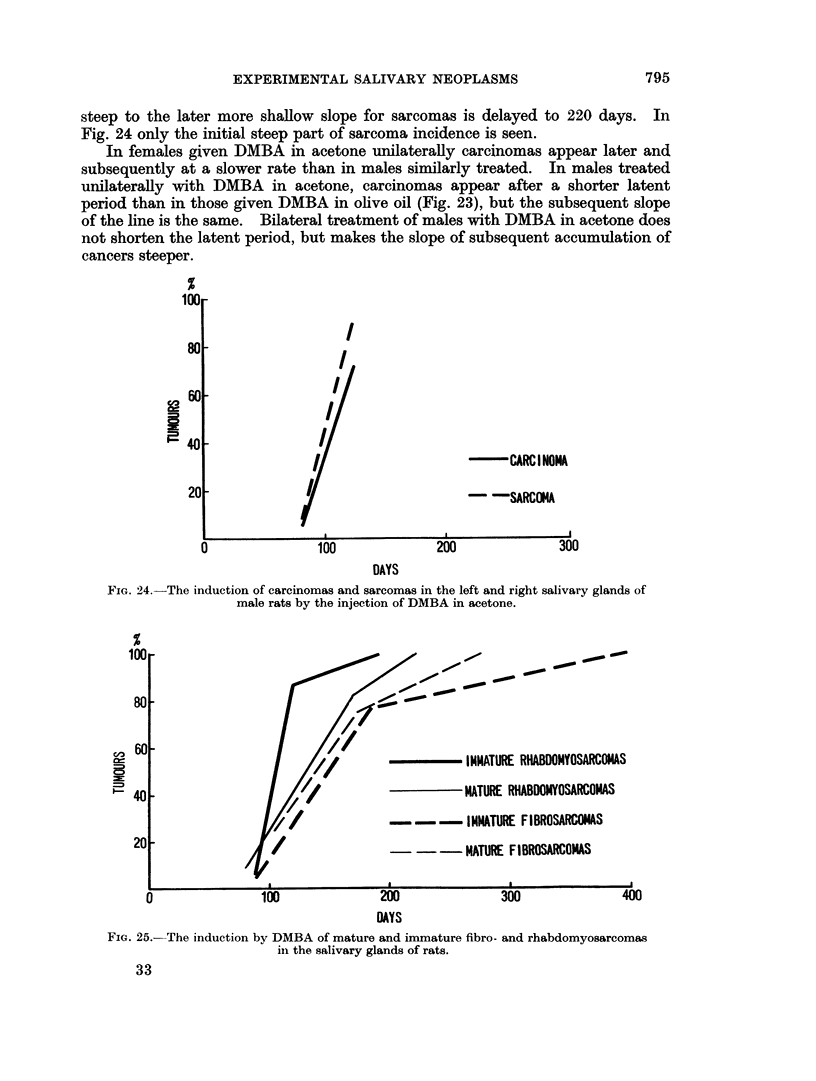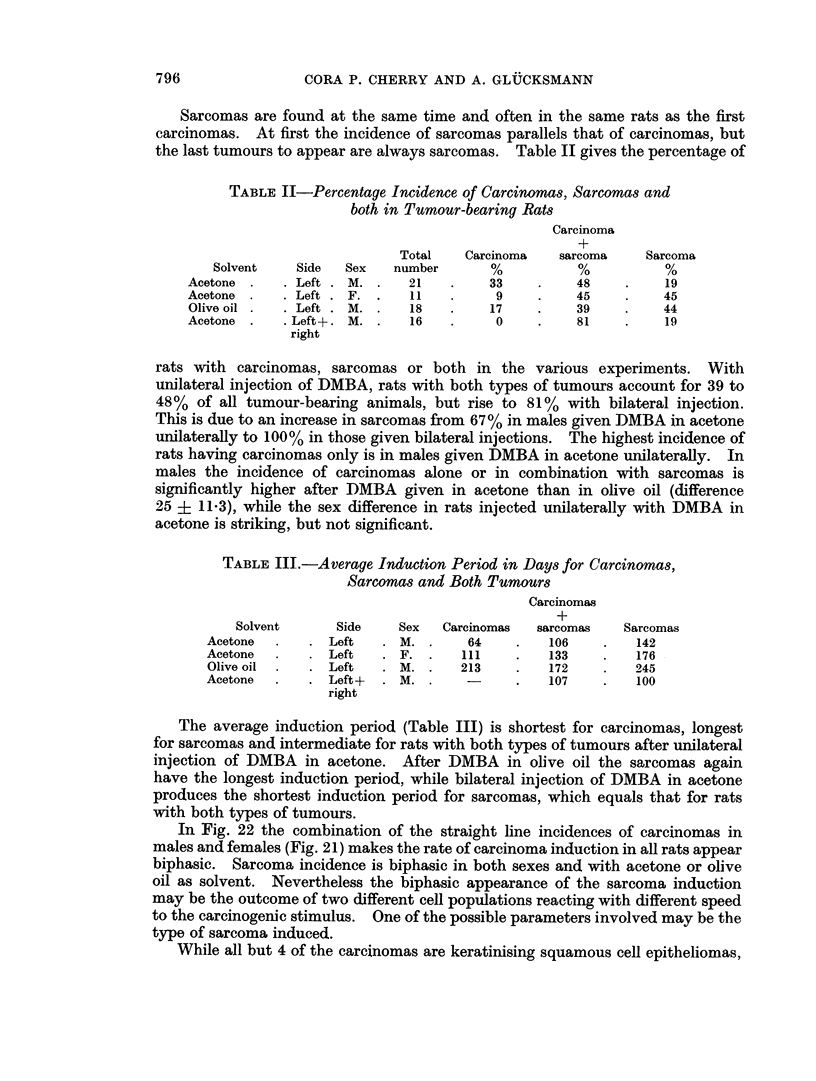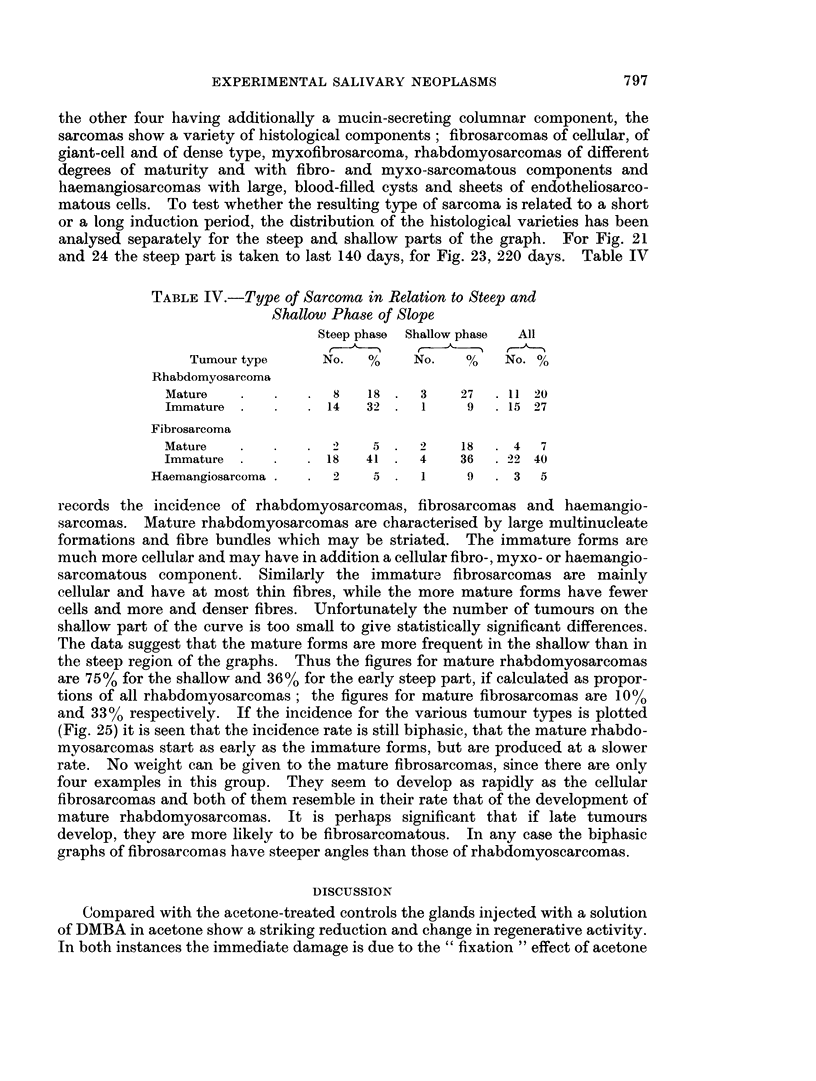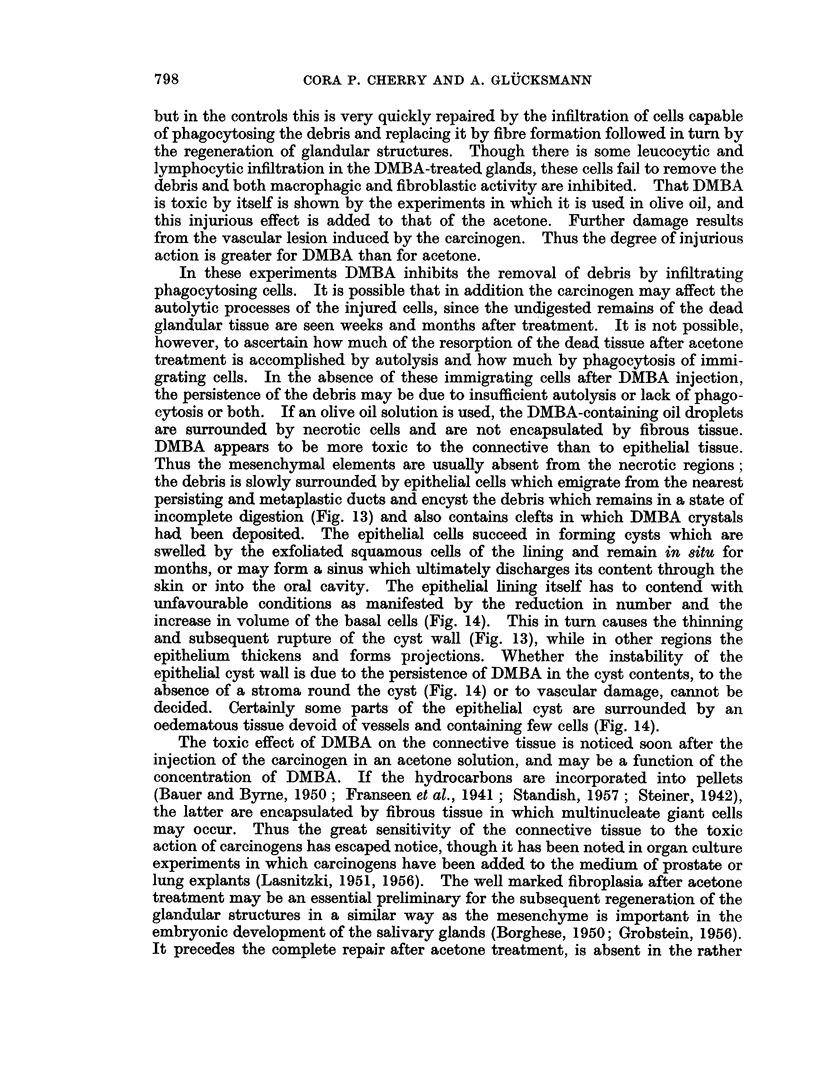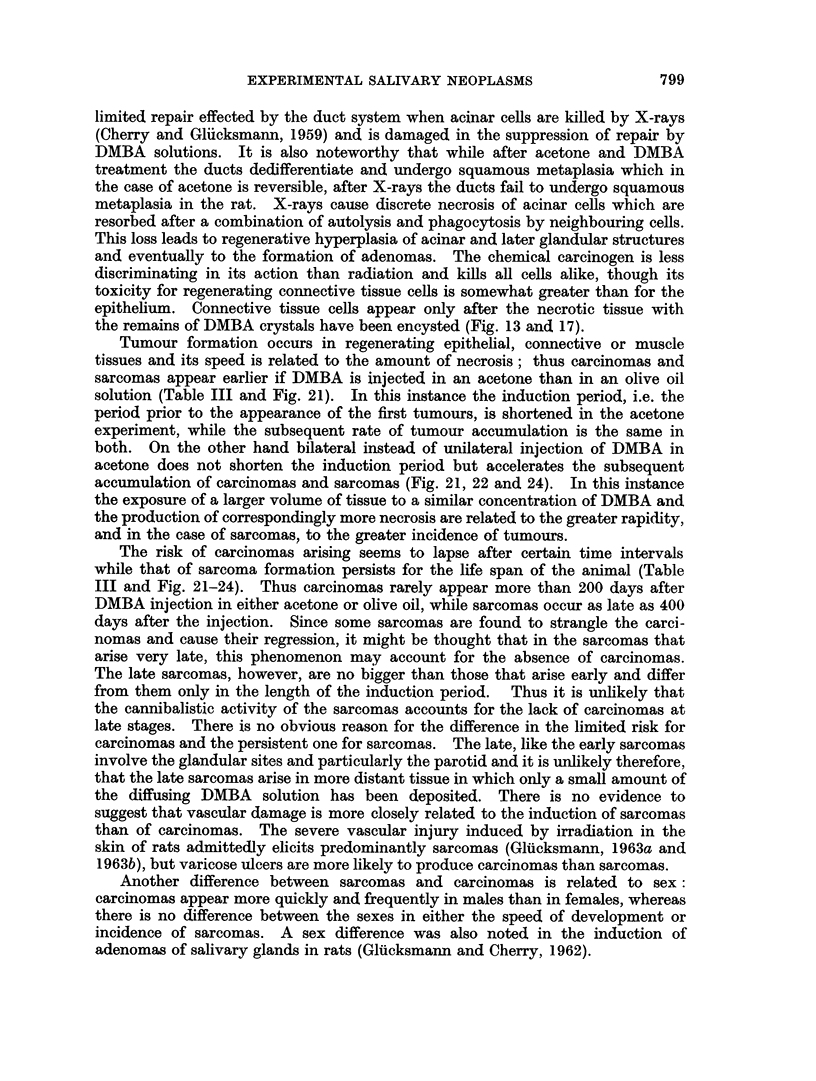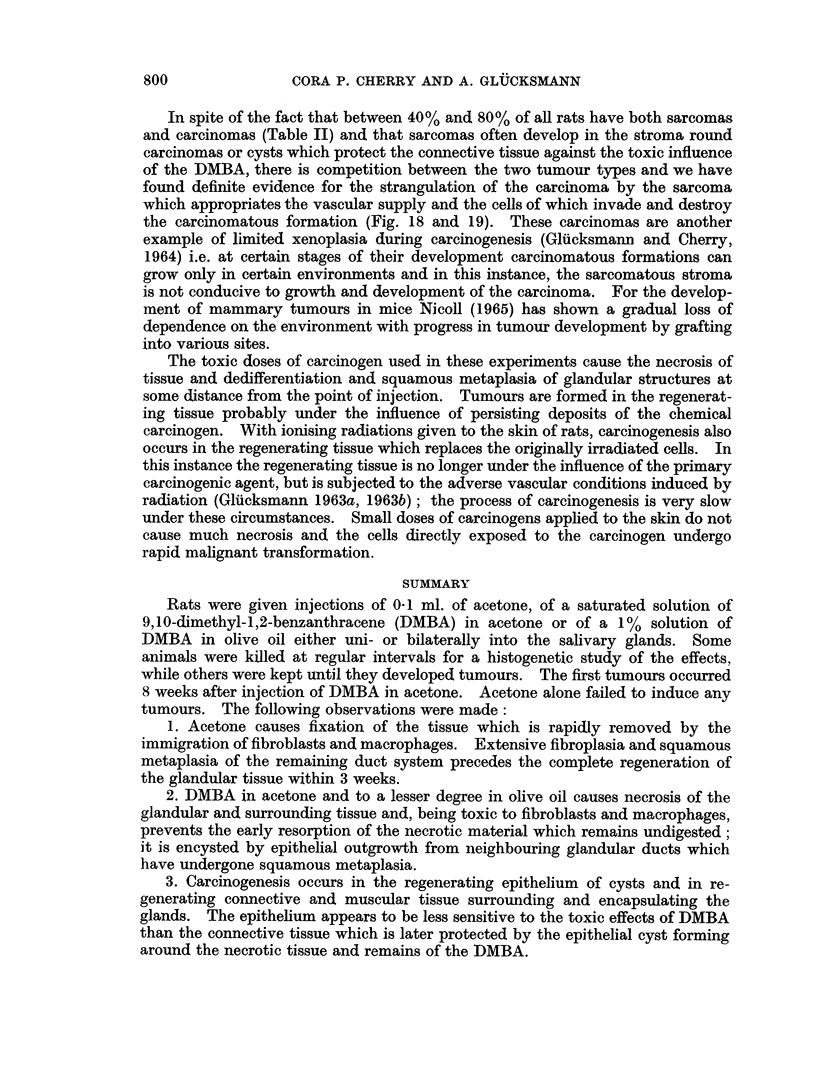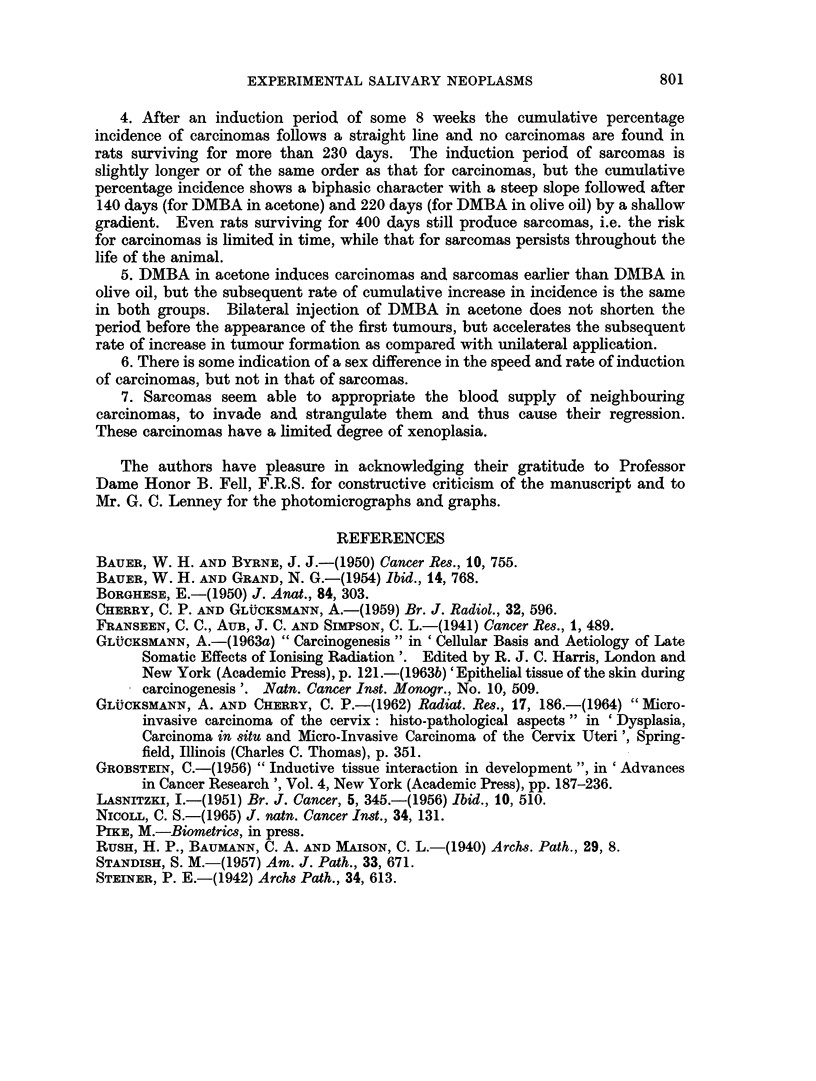# The histogenesis of carcinomas and sarcomas induced in the salivary glands of rats.

**DOI:** 10.1038/bjc.1965.91

**Published:** 1965-12

**Authors:** C. P. Cherry, A. Glücksmann

## Abstract

**Images:**


### THE HISTOGENESIS OF CARCINOMAS AND SARCOMAS INDUCED IN THE SALIVARY GLANDS OF RATS

CORA P. CHERRY* AND A. GLÜCKSMANN†

*From the Strangeways Research Laboratory, Cambridge*

Received for publication July 27, 1965

CARCINOMAS and sarcomas have been induced in the salivary glands of mice, rats and guinea-pigs by the local application of various chemical carcinogens (Rush, Baumann and Maison, 1940 ; Franseen, Aub and Simpson, 1941 ; Steiner, 1942 ; Bauer and Byrne, 1950 ; Bauer and Grand, 1954 ; Standish, 1957), while rabbits have proved to be very resistant and no malignant tumours have been induced in this species (Steiner ; Bauer and Byrne). No true " mixed tumours " as seen in human salivary glands have been reported although Bauer and Byrne found adenomatous structures in a pseudo-cartilage matrix in some tumours. Carcinomas were induced more frequently than sarcomas and generally took a shorter time to develop. Although most commonly the carcinomas were of the squamous cell type, adenoacanthomas and an adenocarcinoma have been reported in mice (Bauer and Byrne, 1950 ; Steiner, 1942). Steiner found some carcino-sarcomas especially in mice under treatment with methylcholanthrene.

In all the experiments with the exception of those of Rush *et al.*, who used corn oil as the solvent, a pellet of cholesterol or wax containing the carcinogen was implanted into the salivary glands. The initial fibrous reaction around the pellet was followed by squamous metaplasia in the adjacent epithelial tissue, the formation of epidermoid cysts and the subsequent development of squamous carcinomas in relation to the walls of the cysts. Rush *et al.*, also found squamous metaplasia of the glandular tissue prior to malignant transformation. Steiner claims that the cells of the acini and ducts undergo metaplasia and thus contribute to tumour formation while Standish maintains that the striated ducts are the source of the carcinomas. Bauer and Byrne state that in their experiments the tumours originated from the cells of the intercalated ducts. Sarcomas probably arose from the stroma within or around the salivary glands (Steiner) or from the proliferating connective tissue around the deposited pellet (Standish). In a previous study on the carcinogenic action of ionizing radiations on the salivary glands, a sex difference was found (Glücksmann and Cherry, 1962) and this observation has led us to investigate the effect of endocrines on the carcinogenesis induced in salivary glands by chemical carcinogens, particularly in male rats, as a corollary to our studies of hormonal effects on the induced carcinogenesis in the cervix and vagina of rats and mice. The present report is concerned with the early effects of the local injection of a chemical carcinogen into the salivary glands of the rat and the histogenesis of the induced tumours.

* Working with a grant from the British Empire Cancer Campaign for Research.
† Gibb Senior Fellow of the British Empire Cancer Campaign for Research.

### MATERIAL AND METHODS

One hundred and twenty-nine male and female black-hooded rats 2 to 5 months old were used for the experiments. 0·1 ml. of a saturated solution of 9,10-dimethyl-1,2-benzanthracene (DMBA) in acetone or of a 1% solution of the carcinogen in olive oil was injected under ether anaesthesia into the salivary gland complex on one or both sides of the neck, in some experiments after and in others without surgical exposure of the salivary glands. An attempt was made to deposit the carcinogen into all 3 salivary glands and thus 0·05 ml. of the solution was injected in an anterior direction into the submandibular and closely applied sublingual glands and the remaining 0·05 ml. was given in a posterior direction to the parotid gland. Control animals received a similar quantity of acetone and the injection was made in the same manner as described above. In the animals given the carcinogen in olive oil, the salivary gland complex on the other side of the neck was used as control and injected with 0·1 ml. of sterile olive oil. Table I gives the details of treatment, sex and number of animals in the different experiments.

TABLE I—*Experimental Procedures and Number of Rats*

| Injection of | Glands | Number of rats | Sex |
|---|---|---|---|
| Acetone | Left | 21 | M. |
| Acetone | Left | 7 | F. |
| Acetone | Left + Right | 14 | M. |
| DMBA in acetone | Left | 33 | M. |
| DMBA in acetone | Left | 14 | F. |
| DMBA in acetone | Left + Right | 20 | M. |
| Olive oil | Right | 20 | M. |
| DMBA in olive oil | Left | 20 | M. |

Rats treated with DMBA in acetone were killed at 1, 3, 5, 7, 10 and 14 days after injection, then at weekly intervals for 5 weeks and thereafter at periods varying from 2 to 8 months whenever tumours or other conditions made it necessary.

Acetone treated controls were killed daily for 14 days, then at 17, 18, 21, 28, 35 and 42 days after injection and thereafter at periods varying from 2 to 8 months.

The rats treated with DMBA in olive oil were killed at intervals ranging from 54 to 477 days after injection when tumours likely to cause death or suffering made it necessary.

At autopsy the salivary gland complex was fixed in Bouin's fluid, dehydrated in routine manner and embedded in paraffin. The blocks were cut serially and every fifth section taken. When tumours were present an attempt was made to identify the salivary glands and when this was possible the tissue was fixed in Bouin's fluid. Tumours were fixed in Zenker-acetic and the blocks were sectioned at 8 $\mu$. Sections were stained with haematoxylin-eosin, the periodic acid-Schiff technique with prior diastase digestion, Southgate's mucicarmine stain, Trevan's alcian blue-basic fuchsin method, van Gieson's method or with carmalum-aniline blue-orange G.

### RESULTS

(a) *Controls : The effect of acetone and olive oil on the salivary glands*

Basically the changes were similar in the 3 glands, but the parotid was more extensively involved than either the submandibular or the sublingual which are more compact and protected by a dense capsule and where the damage tended to be limited to the peripheral parts of the glands.

The acetone diffuses rapidly and causes an almost immediate " fixation " of the glandular tissue which is blanched within a few seconds after injection. A large area of the organ may be involved ; in the affected part the damage is uniform and acini, intercalated and distal excretory ducts and in the submandibular secretory tubules are equally injured. One day after injection the fixed tissue has an eosinophilic appearance in sections stained with haematoxylin and eosin and although nuclei are visible in the cells they stain only faintly with haematoxylin (Fig. 1). The interlobular connective tissue is very oedematous with some leucocytic infiltration which extends into the killed part of the gland. At this stage in the less damaged glandular tissue at the periphery of the fixed lesion, the acini are collapsed and show little or no secretory activity. The collapse of acini leads to an apparent increase in cellularity in this part. The blood vessels are affected to a varying degree, i.e. from fixation of the walls and clotting of the contents to oedematous swelling of the walls and mere dilatation in the less damaged regions.

The injury is followed rapidly by the removal of the dead tissue and regenerative activity in which fibroblasts play a dominant part. The initial oedema is replaced by fibrosis. The less injured glandular parts dedifferentiate to an almost uniform duct-like system which undergoes squamous metaplasia (Fig. 2) and starts to sprout. The " fixed " glandular tissue is infiltrated by fibroblast-like cells which separate the dead acini, and appear to break down and resorb the dead tissue, thus assuming the functions and appearance of small macrophages (Fig. 3) with eosinophilic cytoplasm. As the dead material is removed, fibre formation begins and fills the vacant spaces which are later colonised by the ingrowth of regenerating glandular structures. These shed their cornifying cells and resume differentiation into ducts and later into secretory tubules and acini.

The less damaged glandular tissue outside the fixed lesion begins to regenerate on the 4th day. In some areas the collapsed acini contain degenerate as well as many mitotic cells ; this regenerative activity on the part of injured acini is particularly conspicuous during the second week after injection when normal and abnormal mitotic acinar cells are seen in parts of the gland that are being recolonised by connective tissue cells (Fig. 4). Other acini at the periphery of the fixed lesion dedifferentiate and appear as dilated sacs lined by low cuboidal or flattened epithelium (Fig. 5) ; the intercalated ducts are dilated and the cylindrical cells of the distal excretory ducts lose their characteristic basal striations and change to a low cuboidal or flattened epithelium. The secretory tubules of the submandibular gland also dedifferentiate and are lined by a flattened nonsecretory epithelium. Larger excretory ducts lead into groups of the dedifferentiated and dilated duct-like structures. On the 6th day after injection the cells of the dedifferentiated structures begin to proliferate and form buds from which new acini are formed. These sprouts grow towards the killed part of the gland in the wake of the invading connective tissue cells.

During the process of regeneration some of the dedifferentiated structures undergo squamous metaplasia. This is seen as early as the 4th day and is most marked between the 6th and 8th days when the lumina may become completely occluded by squamous cells. This metaplasia is only temporary and by the 9th day some of the squamous cells and keratinised material are being shed. The exfoliation continues throughout the process of regeneration and eosinophilic debris may be seen in some of the smaller ducts at the periphery of the regenerating gland towards the end of the third week (Fig. 6).

During the second and third weeks after injection, undamaged acini in the submandibular gland may show increased mucin secretion and stain as intensely with mucicarmine, PAS and alcian blue as the acini of the normal sublingual gland.

The continued regeneration from persisting viable acinar cells together with the formation of new acini from the proliferating dedifferentiated structures, repopulate the fixed part of the gland and ultimately restore it to normal. The whole lesion produced by acetone is repaired in about 3 weeks without the formation of a sequester and the only evidence of the previous damage may be a slight fibrosis in the capsule of the gland.

The injection of acetone into the left and right glands produces effects similar to those seen after unilateral application. No tumours have been induced by this treatment and there were no significant abnormalities in the glands of rats kept for 240 days after injection. Acetone injected into the glands spreads to (and fixes) the adjacent muscle. The dead muscle tissue is broken down by infiltrating fibroblast-like cells and on the second day after injection muscular debris is being removed by macrophages and regeneration from myoblasts has begun. By the end of the first week the debris of the dead muscle has disappeared and muscular regeneration is well advanced. No muscular or connective tissue tumours were found either in the rats treated with acetone only, or in those treated with olive oil.

The injection of olive oil into the glands does not cause any " fixation " of tissue. The oil droplets are broken down into smaller units in the course of a granulomatous reaction which persisted for 197 days at least. No tumours or precancerous changes were seen in these glands.

(b) *The histogenesis of carcinomas and sarcomas induced by DMBA*

The injection of DMBA in acetone causes similar changes in the 3 glands but, as with acetone alone, the parotid is the most extensively involved while in the submandibular and sublingual the initial effects are confined mainly to the periphery of the glands.

The initial uniform fixation of the affected part is followed by a well marked oedematous and slight inflammatory cell response in the interlobular and capsular connective tissue, which is seen 1 day after injection (Fig. 7). The DMBA has a toxic effect on the connective tissue and inhibits the fibroblastic response produced by acetone alone. Since the DMBA crystals persist for some weeks at the site of injection as indicated by the clefts left after histological processing (Fig. 11), their toxic influence continues and is evidenced by (1) progressive acinar degeneration and dedifferentiation of acini, tubules and ducts which extends into the " unfixed "

part of the glands; (2) progressive vascular changes leading to swelling and hyalinisation of the walls and narrowing of the lumina; (3) absence of fibroblastic regenerative and phagocytic activity. The fibroblasts are more sensitive to the DMBA than the epithelial cells and instead of invading and removing the dead acinar tissue (Fig. 3) as in the acetone-controls, they die; epithelial cells originating from collapsed, dedifferentiated and metaplastic glandular structures attempt to form an epithelial capsule (Fig. 8) around the fixed tissue which is infiltrated to a very varying degree by lymphocytes and leucocytes. Degeneration of epithelial cells, and leucocytic and round cell infiltration are found also in the dedifferentiated glandular elements which have undergone squamous metaplasia (Fig. 9) and are embedded in an oedematous connective tissue almost devoid of cells and vessels. These environmental conditions cause further degeneration among the encysting epithelial cells (Fig. 10) and even 4 weeks after injection the dead tissue remains undigested and only partly encysted (Fig. 11). The absence of fibroblasts even outside the encapsulating epithelium at this stage is in striking contrast to the acetone controls, where at this period digestion of dead tissue and regeneration is complete (Fig. 12 and Fig. 3, 5, 6). By about the 8th week most of the dead glandular tissue is surrounded by an epithelial capsule (Fig. 13) which may link up with the large and distal parts of the excretory ducts, form a sinus or remain as an enlarging cyst containing exfoliated squamous cells as well as the dead glandular tissue (Fig. 14). The cyst and sinus connect with the collapsed, dedifferentiated glandular elements which are lined by flat or cuboidal epithelium or have undergone squamous metaplasia. In some regions the cysts are lined by a few layers of squamous epithelium which is surrounded by oedematous connective tissue almost devoid of cells (Fig. 14), while elsewhere the epithelium is very hyperplastic, forms projections and is enclosed by a more cellular stroma (Fig. 13). From these excrescences carcinomas develop as early as 56 days after injection of DMBA, though in some rats tumours appeared as late as 174 days after the same treatment. The tumours are usually squamous cell carcinomas which arise in many foci, are usually surrounded by cellular and thin-fibred stroma (Fig. 15) and expand locally into the glands, the surrounding connective tissue, muscle and lymph nodes. Since the animals were killed at the first sign of local tumour formation, metastatic spread of the tumours was not found.

At about 8 weeks, when the first tumours are seen, the vascular changes in the form of hyalinisation of the walls and narrowing of the lumina are very conspicuous (Fig. 16).

At some distance from the DMBA-deposits and particularly when separated from them by epithelial layers, the oedematous connective tissue is repopulated slowly by fibroblasts, many of which degenerate. By the 8th week some abnormally large fibroblasts are seen close to the epithelial cysts (Fig. 17) and some of them undergo normal or abnormal divisions. These cells are probably the stem cells for the sarcomatous transformation that occurs frequently around the epithelial and carcinomatous projections from the cyst walls. The sarcomas are usually very cellular, thin-fibred and grow rapidly. They may deprive the associated carcinomas of their blood supply and thus cause their regression by "strangulation". Such carcinomatous formations lose their basal layers (Fig. 18 and 19) and are represented finally by keratinised remains in the centre of the sarcomatous tissue.

### EXPLANATION OF PLATES

FIG. 1.—Parotid, 1 day after injection of acetone. Killed (n) glandular tissue borders the oedematous interlobular connective tissue (ct) and surviving but collapsed (c) gland. H. & E. ×65.

FIG. 2.—Parotid, 4 days after injection of acetone. Dead (n) glandular tissue is surrounded and invaded by fibroblast-like cells which accumulate also around the persisting, dedifferentiated ducts (d) which have undergone squamous metaplasia. H. & E. ×80.

FIG. 3.—Parotid, 8 days after injection of acetone. The fibroblast-like cells invading the glandular tissue (n) are phagocytosing the debris and at the periphery fibroplasia (f) has begun. H. & E. ×120.

FIG. 4.—Parotid, 11 days after injection of acetone. Invasion of the partly dead glandular tissue (n) by macrophages (Mc) ; less injured cells attempt mitosis, usually resulting in abnormal divisions (m). H. & E. ×240.

FIG. 5.—Parotid, 8 days after injection of acetone. Persisting acini and ducts have dedifferentiated to squamous formations and are encircled by many fibroblasts and small macrophages. H. & E. ×120.

FIG. 6.—Sublingual, 21 days after injection of acetone. Marked regenerative activity at the periphery of the gland with exfoliation of cornified material into the lumina of ducts (d), resumption of the cylindrical or cuboidal shape of lining cells and the budding of terminal ducts (b). Numerous fibroblasts (f) are present near the regenerating glandular structures. H. & E. ×135.

FIG. 7.—Sublingual, 1 day after injection of DMBA in acetone. The peripheral part of the gland is dead (n) and borders the oedematous capsular connective tissue (ct) invaded by some leucocytes.

FIG. 8.—Parotid, 8 days after injection of DMBA in acetone. Persisting necrotic glandular tissue (n) is being encysted by epithelium (e) which has migrated from neighbouring dedifferentiated ducts. Note the accumulation of dying leucocytes (1) and the oedematous connective tissue (ct). H. & E. ×120.

FIG. 9.—Submandibular, 10 days after injection of DMBA in acetone. Compare with Fig. 5. Degenerating epithelial cells, leucocytes and lymphocytes are seen in the dedifferentiated squamous celled structures which are embedded in oedematous connective tissue. Note the swelling and hyalinisation of the wall of the adjacent vessel (v). H. & E. ×120.

FIG. 10.—Parotid, 21 days after injection of DMBA in acetone. The necrotic (n) glandular tissue with infiltrating and dying leuco- and lymphocytes is being encapsulated by epithelial cells (e) which vary greatly in size. Some of them are degenerating. The surrounding connective tissue (ct) is oedematous and contains few cells, some of which are degenerating H. & E. ×120.

Fig. 11.—Parotid, 28 days after injection of DMBA in acetone. Clefts (cl) in the necrotic glandular tissue indicate the deposits of DMBA crystals dissolved during the histological processing. The debris (n) containing degenerating round cells is partially encysted by squamous epithelium (e). H. & E. ×65.

FIG. 12.—Parotid, 28 days after injection of DMBA in acetone. The connective tissue (ct) outside the partially encysted debris (n) is oedematous and contains very few cells, some of which are degenerating. Compare with Fig. 3, 5 and 6. H. & E. ×135.

FIG. 13.—Parotid, 58 days after injection of DMBA in acetone. An almost closed epithelial cyst encapsulates necrotic glandular remains (n) as well as exfoliated squamous cells (s). The epithelial lining varies in thickness (cf. Fig. 14) and in places forms extensive projections (p) into the cellular stroma. H. & E. ×25.

FIG. 14.—Part of the wall of the cyst shown in Fig. 13 at higher magnification. The epithelium is thin, the basal layer contains relatively few, but enlarged cells and borders on oedematous stroma almost devoid of cells. Undigested remains of necrotic gland (n) tissue and exfoliated squamous cells (s) are seen in the cyst. H. & E. ×135.

FIG. 15.—Squamous cell carcinoma replacing the salivary glands, 111 days after injection of DMBA in acetone. The well differentiated tumour foci are surrounded by a cellular thin-fibred stroma. Van Gieson, ×75.

FIG. 16.—Vascular changes in the parotid, 56 days after injection of DMBA in acetone. The lumina of the vessels are narrowed by a severe swelling and hyalinisation of the walls and a perivascular infiltration is present. H. & E. ×135.

FIG. 17.—An area outside the cyst shown in Fig. 13. Only few but large fibroblasts are present next to lymphocytes. In the 4 dividing fibroblasts the mitosis is obviosly abnormal (am) in 3, and normal in 1 (m). H. & E. ×205.

FIG. 18.—Sarcoma and carcinoma in salivary glands, 111 days after injection of DMBA in acetone. The sarcoma is growing and eroding (a) the basal layers of the carcinomatous foci which have been reduced to mere keratinised remains (k). H. & E. ×75.

FIG. 19.—Sarcoma and carcinoma in salivary glands, 103 days after injection of DMBA in acetone. The carcinomatous foci appear compressed, are losing or have lost their basal layers and basement membrane (a) and some are reduced to keratinised remains (k). H. & E. ×140.

FIG. 20.—A fairly mature rhabdomyosarcoma in the muscles surrounding the salivary glands, 105 days after injection of DMBA in acetone. H. & E ×265.

1

3

2

5

6

4

Cherry and Glücksmann.

7

8

10

9

11

Cherry and Glücksmann.

12

13

14

Cherry and Glücksmann.

15

16

17

Cherry and Glücksmann.

18

19

20

Cherry and Glücksmann.

Sarcomas arise also independently of the cysts, in the connective tissue surrounding the glands and also in the regenerating regions of striated muscles close to the glands. The injection of DMBA in acetone causes " fixation " of muscle which, however, persists without eliciting a cellular reaction for at least 87 days. The necrotic and somewhat waxy muscle fibres remain undigested by phagocytic activity. In the periphery of the lesion the muscle fibres are separated by oedema, are degenerate, and digested by phagocytes, and more peripherally situated fibres are beginning to regenerate. These regenerating fibres undergo malignant transformation if they come under the toxic influence of persistent DMBA deposits and give rise to rhabdomyosarcomas of varying degree of maturity (Fig. 20). DMBA in oil does not cause any fixation of the muscle, but the toxic effect of adjacent DMBA causes the fibres to degenerate and regenerative attempts lead to the formation of rhabdomyosarcomas.

(c) *Tumour incidence in relation to sex, solvent of the carcinogen and uni- or bilateral treatment of glands*

The rate of tumour induction in male and female rats is given in Fig. 21 and 22 for unilateral injection of DMBA in acetone, in Fig. 23 for male rats injected unilaterally with DMBA in olive oil and in Fig. 24 for males injected bilaterally with DMBA in acetone. The percentages of rats having carcinomas or sarcomas are plotted ; many animals have both tumours and appear in both curves. Rats are considered at risk if surviving for at least 56 days, i.e. the time when the first cancers were found. The number of animals at risk are 22 males and 12 females (Fig. 21 and 22), 19 males (Fig. 23) and 18 males (Fig. 24), a total of 71 rats.

For carcinomas the time between injection of DMBA and the appearance of the first tumours is longer in females than in males (Fig. 21) and the slope of cumulative tumour incidence in females is less steep than in males treated in the same manner. For sarcomas the sex difference in induction time and cumulative rate is negligible. While a straight line represents the cumulative incidence of

FIG. 21.—The induction of carcinomas and sarcomas in the left salivary glands of male and female rats by the injection of DMBA in acetone.

carcinomas in males and females (Fig. 21 ; for statistical evaluation of the data, see Pike, 1965) the graph for sarcoma incidence in both sexes is biphasic. The initial slope parallels that for carcinomas, while the later slope is much shallower. The change occurs at about 140 days. A similar phenomenon is seen in Fig. 23 : a straight line for carcinoma induction, but a biphasic graph for sarcomas. In this instance the latent period between injection of DMBA and appearance of carcinomas and sarcomas is longer than in Fig. 21 and the change from the early

FIG. 22.—The induction of carcinomas and sarcomas in the left salivary glands of rats (male plus female) by the injection of DMBA in acetone.

FIG. 23.—The induction of carcinomas and sarcomas in the left salivary glands of male rats by the injection of DMBA in olive oil.

steep to the later more shallow slope for sarcomas is delayed to 220 days. In Fig. 24 only the initial steep part of sarcoma incidence is seen.

In females given DMBA in acetone unilaterally carcinomas appear later and subsequently at a slower rate than in males similarly treated. In males treated unilaterally with DMBA in acetone, carcinomas appear after a shorter latent period than in those given DMBA in olive oil (Fig. 23), but the subsequent slope of the line is the same. Bilateral treatment of males with DMBA in acetone does not shorten the latent period, but makes the slope of subsequent accumulation of cancers steeper.

FIG. 24.—The induction of carcinomas and sarcomas in the left and right salivary glands of male rats by the injection of DMBA in acetone.

FIG. 25.—The induction by DMBA of mature and immature fibro- and rhabdomyosarcomas in the salivary glands of rats.

Sarcomas are found at the same time and often in the same rats as the first carcinomas. At first the incidence of sarcomas parallels that of carcinomas, but the last tumours to appear are always sarcomas. Table II gives the percentage of

TABLE II—*Percentage Incidence of Carcinomas, Sarcomas and both in Tumour-bearing Rats*

| Solvent | Side | Sex | Total number | Carcinoma % | Carcinoma + sarcoma % | Sarcoma % |
|---|---|---|---|---|---|---|
| Acetone | Left | M. | 21 | 33 | 48 | 19 |
| Acetone | Left | F. | 11 | 9 | 45 | 45 |
| Olive oil | Left | M. | 18 | 17 | 39 | 44 |
| Acetone | Left + right | M. | 16 | 0 | 81 | 19 |

rats with carcinomas, sarcomas or both in the various experiments. With unilateral injection of DMBA, rats with both types of tumours account for 39 to 48% of all tumour-bearing animals, but rise to 81% with bilateral injection. This is due to an increase in sarcomas from 67% in males given DMBA in acetone unilaterally to 100% in those given bilateral injections. The highest incidence of rats having carcinomas only is in males given DMBA in acetone unilaterally. In males the incidence of carcinomas alone or in combination with sarcomas is significantly higher after DMBA given in acetone than in olive oil (difference $25 \pm 11{\cdot}3$), while the sex difference in rats injected unilaterally with DMBA in acetone is striking, but not significant.

TABLE III.—*Average Induction Period in Days for Carcinomas, Sarcomas and Both Tumours*

| Solvent | Side | Sex | Carcinomas | Carcinomas + sarcomas | Sarcomas |
|---|---|---|---|---|---|
| Acetone | Left | M. | 64 | 106 | 142 |
| Acetone | Left | F. | 111 | 133 | 176 |
| Olive oil | Left | M. | 213 | 172 | 245 |
| Acetone | Left + right | M. | — | 107 | 100 |

The average induction period (Table III) is shortest for carcinomas, longest for sarcomas and intermediate for rats with both types of tumours after unilateral injection of DMBA in acetone. After DMBA in olive oil the sarcomas again have the longest induction period, while bilateral injection of DMBA in acetone produces the shortest induction period for sarcomas, which equals that for rats with both types of tumours.

In Fig. 22 the combination of the straight line incidences of carcinomas in males and females (Fig. 21) makes the rate of carcinoma induction in all rats appear biphasic. Sarcoma incidence is biphasic in both sexes and with acetone or olive oil as solvent. Nevertheless the biphasic appearance of the sarcoma induction may be the outcome of two different cell populations reacting with different speed to the carcinogenic stimulus. One of the possible parameters involved may be the type of sarcoma induced.

While all but 4 of the carcinomas are keratinising squamous cell epitheliomas, the other four having additionally a mucin-secreting columnar component, the sarcomas show a variety of histological components ; fibrosarcomas of cellular, of giant-cell and of dense type, myxofibrosarcoma, rhabdomyosarcomas of different degrees of maturity and with fibro- and myxo-sarcomatous components and haemangiosarcomas with large, blood-filled cysts and sheets of endotheliosarcomatous cells. To test whether the resulting type of sarcoma is related to a short or a long induction period, the distribution of the histological varieties has been analysed separately for the steep and shallow parts of the graph. For Fig. 21 and 24 the steep part is taken to last 140 days, for Fig. 23, 220 days. Table IV

TABLE IV.—*Type of Sarcoma in Relation to Steep and Shallow Phase of Slope*

| Tumour type | Steep phase No. | Steep phase % | Shallow phase No. | Shallow phase % | All No. | All % |
|---|---|---|---|---|---|---|
| Rhabdomyosarcoma | | | | | | |
| Mature | 8 | 18 | 3 | 27 | 11 | 20 |
| Immature | 14 | 32 | 1 | 9 | 15 | 27 |
| Fibrosarcoma | | | | | | |
| Mature | 2 | 5 | 2 | 18 | 4 | 7 |
| Immature | 18 | 41 | 4 | 36 | 22 | 40 |
| Haemangiosarcoma | 2 | 5 | 1 | 9 | 3 | 5 |

records the incidence of rhabdomyosarcomas, fibrosarcomas and haemangiosarcomas. Mature rhabdomyosarcomas are characterised by large multinucleate formations and fibre bundles which may be striated. The immature forms are much more cellular and may have in addition a cellular fibro-, myxo- or haemangiosarcomatous component. Similarly the immature fibrosarcomas are mainly cellular and have at most thin fibres, while the more mature forms have fewer cells and more and denser fibres. Unfortunately the number of tumours on the shallow part of the curve is too small to give statistically significant differences. The data suggest that the mature forms are more frequent in the shallow than in the steep region of the graphs. Thus the figures for mature rhabdomyosarcomas are 75% for the shallow and 36% for the early steep part, if calculated as proportions of all rhabdomyosarcomas ; the figures for mature fibrosarcomas are 10% and 33% respectively. If the incidence for the various tumour types is plotted (Fig. 25) it is seen that the incidence rate is still biphasic, that the mature rhabdomyosarcomas start as early as the immature forms, but are produced at a slower rate. No weight can be given to the mature fibrosarcomas, since there are only four examples in this group. They seem to develop as rapidly as the cellular fibrosarcomas and both of them resemble in their rate that of the development of mature rhabdomyosarcomas. It is perhaps significant that if late tumours develop, they are more likely to be fibrosarcomatous. In any case the biphasic graphs of fibrosarcomas have steeper angles than those of rhabdomyoscarcomas.

### DISCUSSION

Compared with the acetone-treated controls the glands injected with a solution of DMBA in acetone show a striking reduction and change in regenerative activity. In both instances the immediate damage is due to the " fixation " effect of acetone but in the controls this is very quickly repaired by the infiltration of cells capable of phagocytosing the debris and replacing it by fibre formation followed in turn by the regeneration of glandular structures. Though there is some leucocytic and lymphocytic infiltration in the DMBA-treated glands, these cells fail to remove the debris and both macrophagic and fibroblastic activity are inhibited. That DMBA is toxic by itself is shown by the experiments in which it is used in olive oil, and this injurious effect is added to that of the acetone. Further damage results from the vascular lesion induced by the carcinogen. Thus the degree of injurious action is greater for DMBA than for acetone.

In these experiments DMBA inhibits the removal of debris by infiltrating phagocytosing cells. It is possible that in addition the carcinogen may affect the autolytic processes of the injured cells, since the undigested remains of the dead glandular tissue are seen weeks and months after treatment. It is not possible, however, to ascertain how much of the resorption of the dead tissue after acetone treatment is accomplished by autolysis and how much by phagocytosis of immigrating cells. In the absence of these immigrating cells after DMBA injection, the persistence of the debris may be due to insufficient autolysis or lack of phagocytosis or both. If an olive oil solution is used, the DMBA-containing oil droplets are surrounded by necrotic cells and are not encapsulated by fibrous tissue. DMBA appears to be more toxic to the connective than to epithelial tissue. Thus the mesenchymal elements are usually absent from the necrotic regions ; the debris is slowly surrounded by epithelial cells which emigrate from the nearest persisting and metaplastic ducts and encyst the debris which remains in a state of incomplete digestion (Fig. 13) and also contains clefts in which DMBA crystals had been deposited. The epithelial cells succeed in forming cysts which are swelled by the exfoliated squamous cells of the lining and remain *in situ* for months, or may form a sinus which ultimately discharges its content through the skin or into the oral cavity. The epithelial lining itself has to contend with unfavourable conditions as manifested by the reduction in number and the increase in volume of the basal cells (Fig. 14). This in turn causes the thinning and subsequent rupture of the cyst wall (Fig. 13), while in other regions the epithelium thickens and forms projections. Whether the instability of the epithelial cyst wall is due to the persistence of DMBA in the cyst contents, to the absence of a stroma round the cyst (Fig. 14) or to vascular damage, cannot be decided. Certainly some parts of the epithelial cyst are surrounded by an oedematous tissue devoid of vessels and containing few cells (Fig. 14).

The toxic effect of DMBA on the connective tissue is noticed soon after the injection of the carcinogen in an acetone solution, and may be a function of the concentration of DMBA. If the hydrocarbons are incorporated into pellets (Bauer and Byrne, 1950 ; Franseen *et al.*, 1941 ; Standish, 1957 ; Steiner, 1942), the latter are encapsulated by fibrous tissue in which multinucleate giant cells may occur. Thus the great sensitivity of the connective tissue to the toxic action of carcinogens has escaped notice, though it has been noted in organ culture experiments in which carcinogens have been added to the medium of prostate or lung explants (Lasnitzki, 1951, 1956). The well marked fibroplasia after acetone treatment may be an essential preliminary for the subsequent regeneration of the glandular structures in a similar way as the mesenchyme is important in the embryonic development of the salivary glands (Borghese, 1950; Grobstein, 1956). It precedes the complete repair after acetone treatment, is absent in the rather limited repair effected by the duct system when acinar cells are killed by X-rays (Cherry and Glücksmann, 1959) and is damaged in the suppression of repair by DMBA solutions. It is also noteworthy that while after acetone and DMBA treatment the ducts dedifferentiate and undergo squamous metaplasia which in the case of acetone is reversible, after X-rays the ducts fail to undergo squamous metaplasia in the rat. X-rays cause discrete necrosis of acinar cells which are resorbed after a combination of autolysis and phagocytosis by neighbouring cells. This loss leads to regenerative hyperplasia of acinar and later glandular structures and eventually to the formation of adenomas. The chemical carcinogen is less discriminating in its action than radiation and kills all cells alike, though its toxicity for regenerating connective tissue cells is somewhat greater than for the epithelium. Connective tissue cells appear only after the necrotic tissue with the remains of DMBA crystals have been encysted (Fig. 13 and 17).

Tumour formation occurs in regenerating epithelial, connective or muscle tissues and its speed is related to the amount of necrosis ; thus carcinomas and sarcomas appear earlier if DMBA is injected in an acetone than in an olive oil solution (Table III and Fig. 21). In this instance the induction period, i.e. the period prior to the appearance of the first tumours, is shortened in the acetone experiment, while the subsequent rate of tumour accumulation is the same in both. On the other hand bilateral instead of unilateral injection of DMBA in acetone does not shorten the induction period but accelerates the subsequent accumulation of carcinomas and sarcomas (Fig. 21, 22 and 24). In this instance the exposure of a larger volume of tissue to a similar concentration of DMBA and the production of correspondingly more necrosis are related to the greater rapidity, and in the case of sarcomas, to the greater incidence of tumours.

The risk of carcinomas arising seems to lapse after certain time intervals while that of sarcoma formation persists for the life span of the animal (Table III and Fig. 21–24). Thus carcinomas rarely appear more than 200 days after DMBA injection in either acetone or olive oil, while sarcomas occur as late as 400 days after the injection. Since some sarcomas are found to strangle the carcinomas and cause their regression, it might be thought that in the sarcomas that arise very late, this phenomenon may account for the absence of carcinomas. The late sarcomas, however, are no bigger than those that arise early and differ from them only in the length of the induction period. Thus it is unlikely that the cannibalistic activity of the sarcomas accounts for the lack of carcinomas at late stages. There is no obvious reason for the difference in the limited risk for carcinomas and the persistent one for sarcomas. The late, like the early sarcomas involve the glandular sites and particularly the parotid and it is unlikely therefore, that the late sarcomas arise in more distant tissue in which only a small amount of the diffusing DMBA solution has been deposited. There is no evidence to suggest that vascular damage is more closely related to the induction of sarcomas than of carcinomas. The severe vascular injury induced by irradiation in the skin of rats admittedly elicits predominantly sarcomas (Glücksmann, 1963*a* and 1963*b*), but varicose ulcers are more likely to produce carcinomas than sarcomas.

Another difference between sarcomas and carcinomas is related to sex : carcinomas appear more quickly and frequently in males than in females, whereas there is no difference between the sexes in either the speed of development or incidence of sarcomas. A sex difference was also noted in the induction of adenomas of salivary glands in rats (Glücksmann and Cherry, 1962).

In spite of the fact that between 40% and 80% of all rats have both sarcomas and carcinomas (Table II) and that sarcomas often develop in the stroma round carcinomas or cysts which protect the connective tissue against the toxic influence of the DMBA, there is competition between the two tumour types and we have found definite evidence for the strangulation of the carcinoma by the sarcoma which appropriates the vascular supply and the cells of which invade and destroy the carcinomatous formation (Fig. 18 and 19). These carcinomas are another example of limited xenoplasia during carcinogenesis (Glücksmann and Cherry, 1964) i.e. at certain stages of their development carcinomatous formations can grow only in certain environments and in this instance, the sarcomatous stroma is not conducive to growth and development of the carcinoma. For the development of mammary tumours in mice Nicoll (1965) has shown a gradual loss of dependence on the environment with progress in tumour development by grafting into various sites.

The toxic doses of carcinogen used in these experiments cause the necrosis of tissue and dedifferentiation and squamous metaplasia of glandular structures at some distance from the point of injection. Tumours are formed in the regenerating tissue probably under the influence of persisting deposits of the chemical carcinogen. With ionising radiations given to the skin of rats, carcinogenesis also occurs in the regenerating tissue which replaces the originally irradiated cells. In this instance the regenerating tissue is no longer under the influence of the primary carcinogenic agent, but is subjected to the adverse vascular conditions induced by radiation (Glücksmann 1963*a*, 1963*b*) ; the process of carcinogenesis is very slow under these circumstances. Small doses of carcinogens applied to the skin do not cause much necrosis and the cells directly exposed to the carcinogen undergo rapid malignant transformation.

SUMMARY

Rats were given injections of 0·1 ml. of acetone, of a saturated solution of 9,10-dimethyl-1,2-benzanthracene (DMBA) in acetone or of a 1% solution of DMBA in olive oil either uni- or bilaterally into the salivary glands. Some animals were killed at regular intervals for a histogenetic study of the effects, while others were kept until they developed tumours. The first tumours occurred 8 weeks after injection of DMBA in acetone. Acetone alone failed to induce any tumours. The following observations were made :

1. Acetone causes fixation of the tissue which is rapidly removed by the immigration of fibroblasts and macrophages. Extensive fibroplasia and squamous metaplasia of the remaining duct system precedes the complete regeneration of the glandular tissue within 3 weeks.

2. DMBA in acetone and to a lesser degree in olive oil causes necrosis of the glandular and surrounding tissue and, being toxic to fibroblasts and macrophages, prevents the early resorption of the necrotic material which remains undigested ; it is encysted by epithelial outgrowth from neighbouring glandular ducts which have undergone squamous metaplasia.

3. Carcinogenesis occurs in the regenerating epithelium of cysts and in regenerating connective and muscular tissue surrounding and encapsulating the glands. The epithelium appears to be less sensitive to the toxic effects of DMBA than the connective tissue which is later protected by the epithelial cyst forming around the necrotic tissue and remains of the DMBA.

4. After an induction period of some 8 weeks the cumulative percentage incidence of carcinomas follows a straight line and no carcinomas are found in rats surviving for more than 230 days. The induction period of sarcomas is slightly longer or of the same order as that for carcinomas, but the cumulative percentage incidence shows a biphasic character with a steep slope followed after 140 days (for DMBA in acetone) and 220 days (for DMBA in olive oil) by a shallow gradient. Even rats surviving for 400 days still produce sarcomas, i.e. the risk for carcinomas is limited in time, while that for sarcomas persists throughout the life of the animal.

5. DMBA in acetone induces carcinomas and sarcomas earlier than DMBA in olive oil, but the subsequent rate of cumulative increase in incidence is the same in both groups. Bilateral injection of DMBA in acetone does not shorten the period before the appearance of the first tumours, but accelerates the subsequent rate of increase in tumour formation as compared with unilateral application.

6. There is some indication of a sex difference in the speed and rate of induction of carcinomas, but not in that of sarcomas.

7. Sarcomas seem able to appropriate the blood supply of neighbouring carcinomas, to invade and strangulate them and thus cause their regression. These carcinomas have a limited degree of xenoplasia.

The authors have pleasure in acknowledging their gratitude to Professor Dame Honor B. Fell, F.R.S. for constructive criticism of the manuscript and to Mr. G. C. Lenney for the photomicrographs and graphs.

### REFERENCES

BAUER, W. H. AND BYRNE, J. J.—(1950) *Cancer Res.*, **10**, 755.
BAUER, W. H. AND GRAND, N. G.—(1954) *Ibid.*, **14**, 768.
BORGHESE, E.—(1950) *J. Anat.*, **84**, 303.
CHERRY, C. P. AND GLÜCKSMANN, A.—(1959) *Br. J. Radiol.*, **32**, 596.
FRANSEEN, C. C., AUB, J. C. AND SIMPSON, C. L.—(1941) *Cancer Res.*, **1**, 489.
GLÜCKSMANN, A.—(1963*a*) " Carcinogenesis " in ' Cellular Basis and Aetiology of Late Somatic Effects of Ionising Radiation '. Edited by R. J. C. Harris, London and New York (Academic Press), p. 121.—(1963*b*) ' Epithelial tissue of the skin during carcinogenesis '. *Natn. Cancer Inst. Monogr.*, No. 10, 509.
GLÜCKSMANN, A. AND CHERRY, C. P.—(1962) *Radiat. Res.*, **17**, 186.—(1964) " Micro-invasive carcinoma of the cervix : histo-pathological aspects " in ' Dysplasia, Carcinoma *in situ* and Micro-Invasive Carcinoma of the Cervix Uteri ', Springfield, Illinois (Charles C. Thomas), p. 351.
GROBSTEIN, C.—(1956) " Inductive tissue interaction in development ", in ' Advances in Cancer Research ', Vol. 4, New York (Academic Press), pp. 187–236.
LASNITZKI, I.—(1951) *Br. J. Cancer*, **5**, 345.—(1956) *Ibid.*, **10**, 510.
NICOLL, C. S.—(1965) *J. natn. Cancer Inst.*, **34**, 131.
PIKE, M.—*Biometrics*, in press.
RUSH, H. P., BAUMANN, C. A. AND MAISON, C. L.—(1940) *Archs. Path.*, **29**, 8.
STANDISH, S. M.—(1957) *Am. J. Path.*, **33**, 671.
STEINER, P. E.—(1942) *Archs Path.*, **34**, 613.